# Reversible oropharyngeal dysphagia secondary to cricopharyngeal sphincter achalasia in a patient with myasthenia gravis: a case report

**DOI:** 10.4076/1757-1626-2-6565

**Published:** 2009-08-07

**Authors:** Richard A Rison

**Affiliations:** 1 University of Southern California, Keck School of Medicine, Los Angeles County Medical Center, Presbyterian Intercommunity Hospital, Neurology Consultants Medical Group, 12291 E. Washington Blvd. Suite #303, Whittier, CA, 90606, USA

## Abstract

Bulbar weakness and fatigue resulting in dysphagia and dysarthria is common in myasthenia gravis. In chronic MG it is often assumed that these symptoms herald an exacerbation of the patient's disease and doses of cholinergic agents and immunomodulatory therapies may be increased, along with initiation of plasma exchange. A case is presented in which dysphagia was refractory to standard MG therapy, leading to the subsequent discovery of cricopharyngeal sphincter achalasia as the primary cause of the patient's symptoms rather than an assumed myasthenia gravis exacerbation. The patient's dysphagia resolved after esophageal dilatation. Cricopharyngeal sphincter achalasia is a common disorder producing dysphagia in the elderly and needs to be considered in the evaluation of a myasthenic patient with worsening dysphagia when standard myasthenia gravis therapy fails. Discussion of myasthenia gravis, cholinergic therapy and cricopharyngeal sphincter achalasia is undertaken. Clinicians are encouraged to consider non-neurologic causes of worsening dysphagia in the myasthenic patient.

## Introduction

Myasthenia gravis (MG) is an autoimmune neuromuscular junction disorder that often presents with ocular weakness involving asymmetric ptosis and diplopia. Oropharyngeal weakness also occurs and recurrent dysarthria and dysphagia not uncommonly signal exacerbations in patients with chronic MG. Therapeutic modalities in relapsing disease include cholinergic medication, steroids and other immunosuppressants, along with primary immunomodulatory agents (e.g., Intravenous Immunoglobulin [IvIg]), and plasmapharesis. In the absence of cholinergic crises, it is often assumed that worsening dysphagia and dysarthria is caused by MG itself and secondary non-neurologic causes are forgotten or overlooked. Cricopharyngeal sphincter achalasia (CSA) is a common disorder producing dysphagia in the elderly, and is responsive to a number of therapeutic procedures. A case is presented of a patient with MG with persistent dysphagia thought to be secondary to an MG exacerbation but was found to have CSA instead which responded to esophageal dilatation.

## Case presentation

An 83-year-old Caucasian man (a national of the United States of America) with a nine year history of myasthenia gravis presented with complaints of difficulty swallowing. The patient complained of having trouble swallowing liquids and his pills. He felt as if there were painless secretions in the back of his throat inhibiting swallowing. He also had intermittent coughing and choking. He had had similar symptoms in the past with past MG relapses. There was no associated double vision, droopy eye lids, or extremity weakness, and he had no complaints of shortness of breath. He had been stable on a combination of Mestinon^® ^(60 mg po q 4 hours) and Immuran^® ^(50 mg po bid which he had been on for the last 8-9 years) and was intolerant to past prednisone. His last major myasthenia gravis relapse which required intubation was approximately 8 years ago and he responded well to plasmapharesis at that time. On this current admission there had been no preceding febrile illnesses and no recent vaccinations. His past medical history was significant for diabetes mellitus type 2, degenerative arthritis and cervical spinal stenosis, a past left nephrectomy secondary to prior staghorn calculi, previous lumbar spine surgery, previous cholecystectomy, previous bariatric surgery, and past peptic ulcers with a previous gastrointestinal bleed secondary to past steroid use. In addition to the above, his medication included insulin and lansoprazole.

On examination he was awake and alert with mild dysarthria but no nasal speech patterns. There were no dysconjugate gaze or nystagmoid patterns, nor was there any evidence of ptosis, facial asymmetry, or fatigability. No tongue atrophy, fasciculations, or furrowing was noted, and the patient was able to puff both cheeks adequately. There was no observed use of any accessory respiratory musculature. His motor strength revealed grade 5/5 on the MRC (Medical Research Council) scale in his bilateral upper extremities proximally and distally with a negative "pump handle" test. Lower extremity testing revealed 5/5 strength. Neck flexors and extensors were 5-/5. Reflexes were 1/4 throughout. No coordination abnormalities were identified.

Laboratory investigations revealed a white blood cell count of 7.77 10 × 3/cumm (normal range 4.0-10.8 10 × 3/cumm) without a left shift. C - reactive protein was 0.33 mg/dL (normal range 0.00-0.80 mg/dL). Sodium level was 138 mEq/L (normal range 132-152 mEq/L), potassium was 4.6 mEq/L (normal range 3.4-5.4 mEq/L), blood urea nitrogen was 21 mg/dL (normal range 5-25 mg/dL), creatinine was 1.0 mg/dL (normal range 0.6-1.8 mg/dL), and blood glucose was 309 mg/dL (normal range 70-120 mg/dL). A creatinine kinase level was normal (83 mg/dL). Acetylcholine receptor antibody assay was elevated at 7.94 nM/L (normal less than .03 nM per liter) and smooth muscle antibodies were negative. Vital capacities were all normal (2.65/2.61/2.61 L) along with maximal inspiratory pressure trials (-40/-50/-50 cm H20). A computerized tomography scan of the neck was performed which did not reveal any abnormal mass in the hypopharynx or larynx.

It was thought that the patient was suffering from an MG exacerbation presenting as a predominant dysphagia, and his dose of Mestinon^® ^was subsequently increased along with initiation of IvIg. It was not felt there was any cholinergic crises component secondary to lack of other symptoms commonly seen in this condition. He did not tolerate the IvIg secondary to hypertension so this was stopped. He was subsequently given plasmapharesis but his dysphagia continued.

Unfortunately esophageal manometry was not performed. It was decided however to obtain a barium swallow X-ray which showed a prominent cricopharyngeus sphincter with incomplete relaxation and no evidence any cricopharyngeal bar. The patient was then scheduled for direct laryngoscopy followed by esophagoscopy with mechanical dilation of the cricopharyngeus sphincter under general anesthesia. He tolerated the procedure well and there were no complications.

The patients' swallowing markedly improved and he was discharged home on his same pre-admission medications without any change in dose. He remains stable on his current medication regimen without any required dose adjustments, and is followed as an outpatient.

## Discussion

Myasthenia gravis (MG) is a rare, autoimmune antibody-mediated T cell-dependent neuromuscular junction disorder that often presents with fluctuating and fatigable painless weakness involving specific muscle groups. It has a prevalence rate of approximately 1/5000. The onset of MG is influenced by gender and age in a bimodal fashion. In patients younger than 40, women predominate and in the fifth decade new cases of MG are evenly distributed between men and women. After age 50, new cases of MG are slightly more common in men. The course is variable, and most patients with initial ocular weakness develop bulbar or limb weakness within three years of initial symptom onset. Ocular weakness involving asymmetric ptosis and binocular diplopia is the most typical initial presentation [1]. Early or isolated oropharyngeal weakness is less common, however approximately 20% of patients with MG will present with dysarthria and dysphagia [2]. Recurrent dysarthria and dysphagia not uncommonly signal exacerbations in patients with chronic MG. Therapeutic modalities in relapsing disease include steroids and other immunosuppressants, along with primary immunomodulatory agents (e.g., Intravenous Immunoglobulin [IvIg]), plasmapharesis, and cholinergic agents.

In a chronic MG patient with worsening symptoms, the doses of cholinergic agents are often increased. Cholinergic crisis may develop with excessive dosing of acetylcholinesterase inhibitors in patients with more severe MG. In cholinergic crises increased muscarinic activity generates copious oropharyngeal and bronchial secretions that may obstruct the airway or be aspirated and may simulate dysphagia. Signs of cholinergic crisis include weakness indistinguishable from myasthenic weakness, muscle fasciculations, and symptoms of increased muscarinic activity including bradycardia [1]. In the absence of cholinergic crises, it is often assumed that worsening dysphagia and dysarthria is caused by MG itself and secondary non-neurologic causes are forgotten or overlooked.

The anatomical regulatory mechanisms of dysphagia in neurologic disease are complex (Please see Schaller and colleagues in Reference 3 for an excellent review). Swallowing and gastrointestinal motility depend on coordinated interactions between central and peripheral structures regulating both voluntary and involuntary neuromuscular activity. With regards to central causes (e.g., stroke) exact cortical and brainstem localization is debatable given a dearth of any detailed studies, however a variety of new imaging studies are promising [3].

Cricopharyngeal sphincter achalasia is a common disorder producing dysphagia in the elderly with a variable clinical presentation. Although the exact incidence of cricopharyngeal dysfunction is unknown, the literature reports cricopharyngeal achalasia as the primary cause of or as a contributor to dysphagia in 5-25% of patients being evaluated for clinical symptoms of dysphagia [4]. Cricopharyngeal achalasia may be primary or secondary. Primary cricopharyngeal achalasia implies that the abnormality that leads to the persistent spasm or failure of relaxation of the cricopharyngeus muscle is confined to the muscle, with no underlying neurologic or systemic cause. This primary group can be further subdivided into primary cricopharyngeal achalasia with no underlying cause (i.e., idiopathic) or cricopharyngeal achalasia caused by intrinsic disorders of the cricopharyngeus muscle (e.g., polymyositis, muscular dystrophy, hypothyroidism, or inclusion body myositis [4].

Unfortunately medical therapy for cricopharyngeal dysfunction has been largely refractory to medical management, including therapy with muscle relaxants. Botulinum toxin injection into the cricopharyngeus muscle has recently been explored as a possible therapeutic intervention, but experience is limited [4]. Given this patients' history of neuromuscular disease, these were not considered viable options.

Several surgical approaches may be considered for treatment of cricopharyngeal dysfunction. Although trans-oral and endoscopic approaches have also been advocated with limited experience, the classic approach is the external cricopharyngeal myotomy [4].

The presented patient elected to undergo the less invasive esophageal dilation procedure as previously described. Given the overall rarity of MG, it is unknown whether cricopharyngeal myotomy is superior to esophageal dilation in these patients.

In retrospect, this patient's course including dysphagia characteristics, lack of diurnal fatigability and fluctuation and paucity of other MG-associated symptoms made CSA more likely. The history of previous MG however confused the picture, and this case serves as an important lesson. In myasthenic patients with worsening dysphagia, all clinicians need to consider non-neuromuscular causes.

## Abbreviations

CSA: cricopharyngeal sphincter achalasia; IvIg: intravenous immunoglobulin; MG: myasthenia gravis; MRC: Medical Research Council.

## Consent

Written informed consent was obtained from the patient for publication of this case report. A copy of the written consent is available for review by the Editor-in-Chief of this journal.

## Competing interests

The author declares that they have no competing interests.
